# Pulse Oximetry Screening in Germany—Historical Aspects and Future Perspectives

**DOI:** 10.3390/ijns4020015

**Published:** 2018-04-28

**Authors:** Frank-Thomas Riede, Christian Paech, Thorsten Orlikowsky

**Affiliations:** 1 Department of Paediatric Cardiology, Heart Centre, University of Leipzig, 04289 Leipzig, Strümpellstr. 39, Germany; 2 Department of Neonatology, University Childrens Hospital Aachen, 52072 Aachen, Pauwelsstr. 30, Germany

**Keywords:** critical congenital heart disease, pulse oximetry screening, Germany

## Abstract

In January 2017, pulse oximetry screening was legally implemented in routine neonatal care in Germany. The preceding developments, which were the prerequisite for this step, are described in the specific context of Germany’s health care system. Continued evaluation of the method is imperative and may lead to modifications in the screening protocol, ideally in accordance with the efforts in other countries.

## 1. Historical Aspects: From Clinical Data

The most severe, life-threatening forms of congenital heart disease (CHD) requiring intervention very early in life are termed critical (CCHD). During the last decades of the past century, advances in surgical techniques, as well as in perioperative intensive care medicine, led to an improved survival of neonates with CCHD but further progress is likely to be limited.

Delayed diagnosis of CCHD with its potential of cardiac collapse and even death has long been recognized, but its relative importance increased only with the above-mentioned developments. In Germany, Prof. P. Schneider, the former head of the department of Paediatric Cardiology at the Leipzig Heart Centre, described addressing the postnatal diagnostic gap in CCHD as a challenge not only for paediatric cardiologists but for all involved in perinatal care, thus requiring an interdisciplinary and collaborative approach [1].

The first step was to increase the awareness of the problem by establishing and participating in regional educational programs for paediatricians, neonatologists and midwives, starting in the first years of the last decade. Although data from the literature at that time was very limited, using pulse oximetry screening to reduce the diagnostic gap in CCHD has been informally proposed in light of its well-known advantages (availability, ease of use, non-invasiveness, and low cost) [1,2,3,4].

A survey in Saxony (the federal state in which the Leipzig Heart Centre is situated) revealed that in January 2006, 62% (*n* = 29) of all responding perinatal and neonatal units (*n* = 47; response rate 92%) used pulse oximetry screening regularly. Thus, the prerequisites were ideal to perform a prospective multicentre study on pulse oximetry screening. In the study population, the prenatal detection rate of CCHD was comparatively high (60%). However, there was still a diagnostic gap of 20%, which could be reduced to 4.4% by pulse oximetry screening [5]. Based on the data from this study, the working Groups for Perinatology and Neonatology of the Saxonian Medical Association recommended the implementation of pulse oximetry screening in routine care in October 2009. At around the same time, Tautz et al. published their experience with pulse oximetry screening paralleled by the large series from Norway and Sweden [6,7,8].

**Figure 1:**
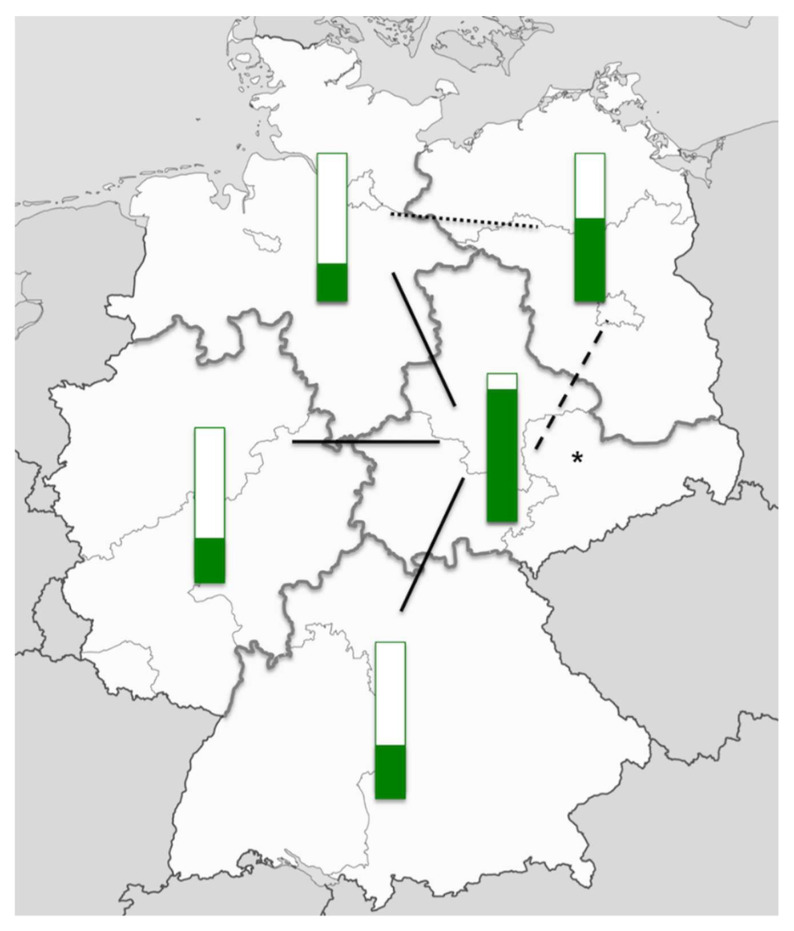
Pulse oximetry screening in Germany in December 2009. Green bars indicate the percentage of perinatal and neonatal units performing pulse oximetry screening as per December 2009 in five regions in Germany. The black lines indicate the level of significance (dotted line: *p* < 0.05, dashed line: *p* < 0.01, solid line, *p* < 0.001). The asterisk indicates the federal state of Saxony. Source of the map: adaptation from svg/2000px-Germany_location_map.svg.png; author: NordNordWest; http://creativecommons.org/licenses/by-sa/3.0/de/legalcode.

In December 2009, a nationwide survey on pulse oximetry screening in Germany was conducted. A questionnaire was sent to all 890 perinatal and neonatal units; the response rate was 29% (*n* = 255). In 46% of the responding units, pulse oximetry screening had already been established. Although, in the majority of cases, it had only been established in the last three years. However, the percentage of units performing pulse oximetry screening showed remarkable regional differences, possibly at least in part related to the regional effects of the activities in Saxony (Figure 1).

**Figure 2:**
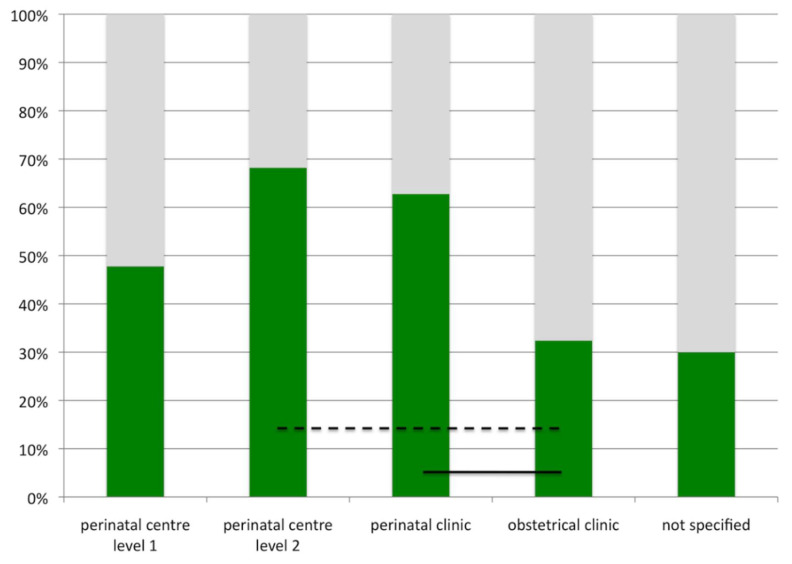
Pulse oximetry screening and level of neonatal care. Green bars indicate percentage of units using pulse oximetry screening as per December 2009 in Germany. Black lines indicate level of significance (dashed line: *p* < 0.01, solid line, *p* < 0.001).

Another interesting finding was to reveal of differences in the use of pulse oximetry screening depending on the level of perinatal care. The latter had been defined in Germany in 2006 in an attempt to centralize the management of risk pregnancies and extreme prematurity (Table 1). Pulse oximetry screening was used less in obstetrical clinics (Figure 2). This may, in part, be explained by the expected low risk profile for pregnancies and neonates in these units. Yet, as existing strategies failed to completely predict CCHD, pulse oximetry screening may be especially useful in these settings.

In light of the increasing body of evidence on the benefits of pulse oximetry screening [11,12], the German Society for Paediatric Cardiology supported the use of pulse oximetry screening in 2011 and formulated a statement with recommendations for practical aspects of its implementation in 2013 [13,14]. Likewise, the German Society for Neonatology and Paediatric intensive care (GNPI) recommended the use of pulse oximetry screening in its guidelines for neonatal care in 2012 [15]. However, the effects of these nonbinding recommendations remained unknown.

## 2. To Legal Regulation

In the nationwide survey of 2009 only 26% of perinatal and neonatal units considered it necessary to have legal regulations in place, in case pulse oximetry screening should be implemented. However, patient representatives had a different view. A group led by the “Bundesverband herzkranke Kinder” (BVHK) initiated the development of a legal regulation.

Germany’s national health care insurance system, introduced in 1883, has been highly regulated. Since 1975, more than 90% of the population have been enrolled in the statutory health insurance, the remaining 10% have been nearly completely covered by private or other health insurance [16]. Thus, regulations concerning the scope of health care services provided by insurers affect almost the entire population.

The regulation of medical care and the implementation of legal requirements on new drugs and methods of treatment by directives are the central tasks of a federal committee (Gemeinsamer Bundesausschuss, G-BA). It was established in January 2004 as the highest decision-making body of the joint self-administration of physicians, dentists, psychotherapists, hospitals and health insurance providers.

Preventive examinations and screening tests in neonates are regulated in the Directive on Early Detection of Diseases in Children up to the age of six years. Currently, each newborn is entitled to three examinations immediately after birth (U1), between the third and tenth day of life (U2), and at the end of (or early after) the neonatal period (fourth to fifth week of life, U3). Extended metabolic screening, hearing screening (since 2009), an ultrasound of the hips and the Brückner test (fundoscopy, since 2016) are also included.

The implementation of a new method requires a formal consultation process and a subsequent positive resolution by the G-BA. The initiation of such a process may be requested by independent members of the G-BA, by health insurers, the Association of Statutory Health Insurance Physicians, the corresponding association of dentists, the German Hospital Society and patient representatives, but not by physicians or medical professional societies.

In September 2012, the group of patient representatives mentioned above submitted an application at the G-BA to initiate a consultation process on pulse oximetry screening, which was granted in November 2012. In June 2013, an independent scientific organisation, the Institute for Quality and Cost Effectiveness in Health Care (Institut für Qualität und Wirtschaftlichkeit im Gesundheitswesen, IQWiG), was commissioned with a detailed analysis of the possible effects of pulse oximetry screening in the current setting of peri- and neonatal care in Germany. The evaluation was mainly based on available data from the literature, but statements from experts, national medical professional associations, and patient representatives were also included. In its final report, published in May 2015, the IQWiG concluded that current evidence suggests a benefit of pulse oximetry screening as an adjunct to the pre-existing diagnostic standard (U1 and U2) with respect to the timely diagnosis of CCHD in neonates [17].

In January 2017, after an internal evaluation of the IQWiG’s report, statements from experts and medical professional associations, a decree of the G-BA was published, announcing the implementation of pulse oximetry screening in routine neonatal care in Germany [18].

**Figure 3:**
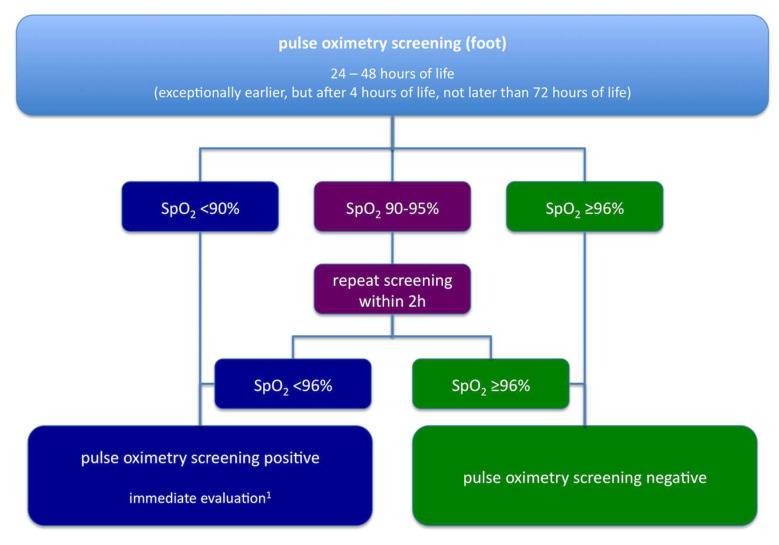
Algorithm for pulse oximetry screening in Germany as recommended by the Common Federal Committee (Gemeinsamer Bundesausschuss, G-BA), modified after [19] by a specialist in paediatrics, ideally with subspecialty training in neonatology/paediatric cardiology.

The algorithm for pulse oximetry screening, as recommended by the G-BA, is shown in Figure 3. In most aspects, it is similar to the one used in the German multicentre study, published by one of the authors in 2010 [5]. However, an important difference lies in the role of echocardiography. According to the current recommendations, echocardiography may be omitted if another cause of hypoxia is found. This approach may be reasonable especially when a positive pulse oximetry screening draws attention to clinical signs suggesting other neonatal pathologies such as pneumonia or sepsis, cases in which echocardiography would only delay appropriate diagnosis and treatment.

## 3. Future Perspectives

As per the decree of the G-BA, the quality and efficacy of pulse oximetry screening in Germany are going to be evaluated after its implementation. By no later than 31 December 2018, an independent scientific institution will be assigned to perform an analysis on the basis of a representative sample to answer a defined set of target parameters (Table 2). However, further aspects might also prove relevant and be included (Table 3).

Screening is definitely better than no screening, and despite the fact that the national screening program will hopefully increase the early detection of CCHD systematically, several controversies will remain with respect to the time of screening, cut off-values, and the follow-up algorithm.

In comparison to the algorithm in the UK, suggesting an early screening before 24 h of age [11], the German, as well as the American algorithm, recommend screening between 24 h and 48 h of age as the “best screening window” [19,20]. The G-BA states that screening may be performed “in exceptional cases at the earliest of four hours after birth” to account for infants discharged from hospital within 24 h after birth. In most published data on screening before 24 h of age, the rate of false positives was up to 10 times higher (0.8% vs. 0.05%) [21]. In this respect, “false positive” means, “false test positive”, i.e., no CCHD detectable in the follow-up. Nevertheless, early screening before 24 h identified more non-cardiac diseases (sepsis, persistent pulmonary hypertension of the newborn) at an early stage before newborns were symptomatic. In the German study, 18 of the 36 newborns with CCHD (50%) showed symptoms before screening [5], which is the very situation that screening aims to prevent. Thus, earlier screening might be desirable from both the neonatologist’s and the paediatric cardiologist’s perspective. However, the impact of pulse oximetry screening on the detection of noncardiac disease has not been evaluated systematically yet. Expanding the spectrum of target parameters for analysis of efficacy of pulse oximetry screening, as determined by the G-BA (Table 2) by additional parameters (Table 3), might provide appropriate data and allow for evidence-based modifications of the screening algorithm so that early screening and a timely diagnosis can be counterbalanced with the false positive rate. Nevertheless, one has to bear in mind that the problem of severe cardiovascular compromise related to CCHD cannot be completely avoided, neither by thorough clinical examination, nor by pulse oximetry, and not even by prenatal diagnosis. This is because for example, in neonates with hypoplastic left heart syndrome or transposition of the great arteries with restrictive or closed foramen ovale, symptoms may occur despite immediate and appropriate treatment.

Modification of the upper time limit for screening might also become necessary. The German algorithm specifies, that the screening may be performed no later than after the 2nd check-up by the paediatrician. Newborns who have already been discharged at that age, receive their standard investigation (U2) from a paediatrician either in his office or at home. This check-up has to be performed between the third and the tenth day of life. In these cases, if early screening has not been performed, substantial risk for falling into a diagnostic gap remains. Furthermore, it would be necessary for all paediatricians to use portable saturation devices for their outpatient visits (U2). For newborns born at home, midwives would have to be trained systematically.

It would be desirable that pulse oximetry screening for the detection of CCHD is recommended for all European countries. A consensus statement on its implementation includes that it should be performed with new-generation equipment that is motion tolerant, after 6 h of life or before discharge from the birthing centre (preferably within 24 h after birth), and should be done in two extremities, the right hand and either foot [22].

The majority of studies used saturations from one post ductal site—either foot. Recently, studies have employed saturations from two sites—the right hand and one foot—giving both pre- and post-ductal saturations. Therefore, rather than a single absolute saturation leading to the test result, two individual values, and also the difference between the two, contribute to the result. Although a systematic review did not identify a significant difference in sensitivity between the two methods [21], this may be explained by the preponderance of single measurement, and further analyses are necessary.

Post hoc analysis of the raw data revealed that post ductal only measurement would miss a small but significant proportion of babies that dual testing would identify [23]. This may become important in Germany considering the birth rate of approximately 800,000 per year.

In contrast to many other studies from the UK and the USA, the cut-off value was chosen to be 96%, following the experiences of the German trial [5]. Other rather big one-step screening studies used 95%. Whether this single point of saturation difference really makes a difference in specificity or false positives remains to be evaluated. However, combining regional or national studies in Europe in meta-analyses, maybe resulting in a common algorithm, is hampered by different thresholds and procedures. For example, all authors recommending a two-site screening use different combinations of thresholds and differences between measurement sites for definition of a positive screening result [7,9,20].

Prenatal echocardiography has the potential to detect virtually all forms of CCHD up to the point where, in single institutions, pulse oximetry screening for CCHD becomes ineffective [24]. Being highly dependent on operator experience and appropriate technical equipment, which are neither widely available, nor will be in the near future, prenatal detection rates in larger regions or countries remain substantially lower [25]. In Germany, the rate of prenatal diagnoses for all CHD has been 12%, ranging from approximately 5 to 68% in CCHD, depending on the type of lesion [26]. In 2013, completion of a four-chamber view has been implemented in routine pregnancy care by the G-BA. It has been estimated that this may ameliorate the prenatal detection rate up to 30–40%, concluding that pulse oximetry screening will be able to substantially contribute to a timely diagnosis of CCDH in the years to come [17].

A recent U.S. study has shown a substantial reduction of mortality from undiagnosed CCHD after the implementation of pulse oximetry screening [27]. In populations where mortality from undiagnosed CCHD was low before the introduction of pulse oximetry screening, a comparable effect might be expected on the reduction of severe morbidity in affected neonates [5,28].

## 4. Conclusions

In the past decade, pulse oximetry screening in Germany has made its way from clinical trials to the statutory introduction into clinical routine as an adjunct to prenatal diagnosis and clinical examination. This will hopefully further improve the prognosis of infants with CCHD. Continuing evaluation of its effectiveness is necessary to allow for modifications of the screening protocol when appropriate.

## Figures and Tables

**Table 1:** Levels of neonatal care in Germany (modified after [9,10]).

Level of Care	Admission Criteria
Perinatal centre level 1	Expected prematurity with a birth weight of <1250 g or a gestational age of <29 weeks triplet pregnancy and gestational age <33 weeks; multiple pregnancy Prenatal diagnosis of any fetal or maternal condition necessitating immediate postnatal intensive care (critical congenital heart disease, diaphragmatic hernia, myelomeningocele, gastroschisis)
Perinatal centre level 2	Expected prematurity with a birth weight of 1250–1499 g or a gestational age of ≥29 to <32 weeks HELLP syndrome Intrauterine growth restriction <3rd percentile Insulin-dependent gestational diabetes with elevated risk for the fetus/newborn
Perinatal clinic	Expected prematurity with a birth weight of ≥1500 g or a gestational age of ≥32 to <36 weeks Intrauterine growth restriction between third and tenth percentile Insulin-dependent gestational diabetes without elevated risk for the fetus/newborn
Obstetrical clinic	Gestational age ≥36 weeks, uncomplicated delivery expected

**Table 2:** Target parameters for the evaluation of pulse oximetry screening after its implementation in Germany as defined by the G-BA [18].

Number and Percentage of Newborns
- having received pulse oximetry screening - with a negative screening result on the first measurement (SpO_2_ ≥ 96%) - with a positive screening result on the first measurement (SpO_2_ < 90%) - with an abnormal result on the first measurement with the need to repeat the test (SpO_2_ 90–95%) - with a negative screening result on the second measurement (SpO_2_ ≥ 96%) - with a positive screening result on the second measurement (SpO_2_ < 96%) - referred to a paediatrician/neonatologist
False positive results
Number of newborns with CCHD detected by pulse oximetry screening
Timing of diagnostic and therapeutic procedures in newborns with CCHD

**Table 3:** Possible additional target parameters for the evaluation of pulse oximetry screening after its implementation in Germany.

Number and Percentage of Newborns
- with a prenatal diagnosis of CCHD - with diagnosis of CCHD based on clinical signs/physical examination before pulse oximetry screening
False negative results
Detection of neonatal diseases in newborns with false positive screening results (with respect to CCHD)
Reasons for not performing pulse oximetry screening in eligible newborns
